# Influencing Factors of Health Technology Assessment to Orphan Drugs: Empirical Evidence in England, Scotland, Canada, and Australia

**DOI:** 10.3389/fpubh.2022.861067

**Published:** 2022-06-17

**Authors:** Na Zhou, Hong Ji, Zheng Li, Jun Hu, Jia-Hua Xie, Yu-Heng Feng, Ni Yuan

**Affiliations:** ^1^Department of Social Medicine, School of Public Health, Dalian Medical University, Dalian, China; ^2^Department of Health Policy and Management, School of Public Health, Peking University, Beijing, China; ^3^Department of Pediatrics, The First Affiliated Hospital of Dalian Medical University, Dalian, China; ^4^Shanghai Medical Products Administration, Shanghai, China

**Keywords:** orphan drugs, health technology assessment (HTA), consistency analysis, correspondence analysis, health insurance reimbursement

## Abstract

This study summarizes the intrinsic criteria for the recommendation of orphan drugs in England, Scotland, Canada, and Australia with the aim of understanding the rationale for the variability in decision-making and to provide a reference for the establishment of criteria in the process of access to health insurance for orphan drugs in different countries and the construction of national uniform criteria. A comparative analysis of 60 health technology assessment (HTA) guidelines of 15 drug-indication pairs appraised by four countries (England, Scotland, Canada, and Australia) from 2017 to 2018 was done, including an in-depth analysis of a case study. Agreement levels were measured using kappa scores. Associations were explored through correspondence analysis. The four countries possess some homogeneity in the assessment, but each has its own preferences. Poor agreement exists between England, Scotland, and Canada (−0.41 < kappa score < 0.192). In the correspondence analysis, England placed more emphasis on treatment methods in terms of control type when making recommendations. Canada and Scotland focused more on trial type with Canada placing more emphasis on phase III and open-label trials and on cost-utility analysis, while Australia was less studied in terms of economic models. Different countries have different goals when establishing HTA decisions for orphan drugs due to their different degrees of orphan drug coverage. Different countries should not only combine their unique values of clinical benefit and cost-effectiveness in the assessment of orphan drugs but also give different weights during the HTA process, after considering account the development of the country itself.

## Introduction

The incidence of rare disease is low; however, rare diseases, which are defined differently in different countries and regions, often endanger health and life. The general principle of the definition of a rare disease is based on the prevalence or number of people with the disease. In the United States (US), a rare disease is defined as a condition that affects fewer than 200,000 people in the country (1). In the European Union (EU), a rare disease is one that affects no more than 1 person in 2,000 people (2). In Australia, a disease is considered rare if it affects <5 in 10,000 people (3).

Health technology assessment (HTA) is a systematic evaluation of the safety, effectiveness, and economic and social aspects of medical technologies *via* an analysis of knowledge about health technologies based on research and practice using the principles and methods of evidence-based medicine (4). In terms of safety, the number of patients with rare diseases is small compared to common diseases. This factor makes it difficult to conduct traditional randomized controlled double-blind trials of orphan drugs to test clinical efficacy, resulting in higher difficulties in the design and implementation of clinical trials, often allowing only single-arm trials, with historical treatment as the default control, or real-world studies to observe the actual clinical effects of orphan drugs after they are launched (5). In terms of economics, the small population of indications for orphan drugs and the fact the cost of diagnosis and treatment of orphan drugs is often high make it difficult to meet the requirements of the traditional incremental cost-effectiveness ratio (ICER) or quality-of-life (QoL) ratio thresholds for evaluation (6). Novartis' Zolgensma, a one-time gene therapy for spinal muscular atrophy tops the list with a price tag of $2.125 million in 2021 (7). Some articles point out that by 2024, orphan drugs are expected to reach $242 billion, capturing one-fifth of worldwide prescription sales, and orphan drugs sale in the world are expected to grow at a compound annual growth rate of 12.3% from 2019 to 2024, which is approximately double the rate foreseen for non-orphan drugs market (8). In addition, other reasons for these high costs, such as the high heterogeneity and low recognition rate of rare diseases that make the diagnosis and treatment difficult and the cost of orphan drugs higher, have been reported. Due to these specificities of HTA for rare diseases, traditional methods, such as randomized controlled trials (RCTs) and cost-effectiveness analyses, may not be applicable to many rare diseases, and orphan drugs are often more prone to uncertainty in cost-effectiveness assessments than common diseases, which makes the process of HTA and health decision making more difficult (9).

It has been shown that an increasing number of countries use HTA to provide relevant information for health technology reimbursement (10). In HTA, cost-effectiveness analysis is one of the commonly used methods for which ICER, especially ICER based on quality-adjusted life years (QALY), has a wide range of applications (11). In the reimbursement of orphan drugs, quality adjusted life year (QALY) is the main QoL indicator for reimbursement decisions, while the treatment of rare diseases can meet the cost effectiveness criteria required for the use of ICER. Relatively speaking, ICER and QALY are not the only factors influencing the reimbursement decision as other social factors that influence the reimbursement decision can be found (12). The National Institute for Health and Clinical Excellence (NICE) gives a relatively generous margin to rare diseases when applying QALY as the threshold for evaluation indicators (13).

At the same time, reimbursement for orphan drugs has encountered challenges, both because clinical evidence may be difficult to collect due to the small number of patients and because their treatments do not meet the cost effectiveness criteria required to use ICERs and are associated with the high price and uncertainty of orphan drugs. More flexible criteria for assessing the clinical value of orphan drugs along with more high-quality clinical evidence for the use of orphan drugs may be needed to increase the likelihood of orphan drug reimbursements by enabling regulators and payers to consistently assess the risks and benefits of orphan drugs in a more efficient manner (12).

Drug reimbursement methods vary across countries ranging from universal health insurance for reimbursement to third-party payment reimbursement by insurance companies, which are separately funded and administered with different reimbursement rates. However, although a proportion of orphan drugs are reimbursed, the proportion of orphan drugs that are reimbursed is still only a small fraction of the total number of orphan drugs (14, 15). In terms of orphan drugs, countries have successively conducted the experience of exploring HTA for rare diseases; however, a need to actively explore a more normative HTA program still exists (9).

After considering well-established assessment systems, similar decision-making criteria, different technical approaches, publicly available HTA reports, and language, four countries were selected for this study: (1) England, (2) Scotland, (3) Australia, and (4) Canada, with the National Institute for Health and Care Excellence (NICE) (16), Scottish Medicines Consortium (SMC) (17), Pharmaceutical Benefits Advisory Committee (PBAC) (18, 19), and Canadian Agency for Drugs and Technologies in Health (CADTH) (20). The purpose of this study was to systematically compare health technology assessment procedures of orphan drugs in these four countries to summarize the intrinsic criteria of orphan drugs recommended in these four countries. The reasons for the variability in access decisions were explained, and countermeasures were proposed to reduce the variability and provide a reference for the establishment of criteria in the process of access to health insurance for orphan drugs. This information will provide a reference for the establishment of criteria for orphan drugs in the health insurance access process.

## Objects and Methods

### Research Subjects

The report was selected from the official websites of the health technology assessment agencies of the four countries, namely NICE, SMC, PBAC, CADTH. NICE produces health technology appraisal guidance to ensure that all National Health Service (NHS) patients have equal access to the most clinically effective and cost-efficient treatments (21). The SMC evaluates drugs to make decisions to recommend, not recommend, or recommend use with restrictions, and drugs approved by the SMC are automatically entered into the Area Drug and Therapeutics Committees (ADTC) reimbursement list. Drugs that are not approved are not entered on the ADTC's reimbursement list, and use of such drugs will be restricted (22). Therapeutic Goods Administration (TGA) is responsible for the marketing approval and orphan drug status of pharmaceutical products in Australia. The Life Saving Drugs Program (LSDP), a free orphan drug program for patients with rare diseases, is available from the LSDP. Before a drug can be offered on the LSDP, it must be evaluated by PBAC as clinically necessary and effective, but it is not recommended for inclusion in the Pharmaceutical Benefits Scheme (PBS) due to unacceptable cost effectiveness (23, 24). In Australia, the reimbursement evaluation of orphan drugs is independent of their cost-effectiveness as long as sufficient evidence of their clinical benefits has been demonstrated (25). The CADTH's recommendation for inclusion on the provincial reimbursement list can be classified as “recommended for inclusion,” “recommended for conditional inclusion,” “not recommended for inclusion at current submission prices,” and “not recommended for inclusion,” based on a combination of clinical effectiveness and economics (26–28).

This study used the orphan drugs queried by NICE in 2017–2018 as a benchmark and queried the corresponding orphan drugs reports for the same drug in three other countries, including a total of 65 orphan drugs. Since this study was expected to explore the factors that influence the relevant authorities in the four countries when conducting orphan drug assessments by having different recommendations for the same drug in different country assessments, orphan drugs that did not have identical assessment results in the four countries were included in this study; thus, 15 drugs were finally included (Table 1).

**Table 1:** HTA assessment for orphan drugs.

**No**.	**Generic name (trade name)**	**Manufacturers**	**Year**	**Whether to review**			
				**(yes, recommended and type of recommendation/yes, not recommended/no)**			
				**NICE** **(England)**	**SMC** **(Scotland)**	**PBAC** **(Australia)**	**CADTH** **(Canada)**
1	Ibrutinib (Imbruvica)	Janssen	2017	Recommended	Not recommended	–	Not recommended
2	Everolimus (Afinitor)	Novartis Pharmaceuticals	2017	Recommended	Recommended	–	Not recommended
3	Daratumumab (Darzalex)	Janssen	2017	Recommended	Restricted use	–	NOT recommended
4	Pegylated liposomal irinotecan (Onivyde)	Shire	2017	Not recommended	NOT recommended	Recommended	–
5	Afatinib (Giotrif)	Boehringer Ingelheim Pty Ltd	2017	Not recommended	Not recommended	Recommended	Recommended
6	Vandetanib (Caprelsa)	Sanofi	2018	Not recommended	Not recommended	Recommended	Recommended
7	Romiplostim (Nplate)	Amgen	2018	Recommended	Restricted use	–	Not recommended
8	Eltrombopag (Revolade)	GlaxoSmithKline	2018	Recommended	Restricted use	Recommended	Not recommended
9	Cabozantinib (Cabometyx)	Ipsen	2018	Recommended	Not recommended	–	–
10	Cenegermin (Oxervate)	Dompé	2018	Not recommended	Not recommended	Recommended	–
11	Cabozantinib (Cometriq)	–	2018	Recommended	Not recommended	–	–
12	Obinutuzumab (Gazyvaro)	Roche	2018	Recommended	Not recommended	Recommended	Recommended
13	Ixazomib with lenalidomide and dexamethasone (Ninlaro)	Takeda	2018	Recommended	Not recommended	Recommended	Not recommended
14	Pirfenidone (Esbriet)	Roche	2018	Recommended	Restricted use	Recommended	Not recommended
15	Lenvatinib with everolimus (Kisplyx)	Eisai	2018	Recommended	Recommended	Recommended	Not recommended

*“-” Reports on health technology assessment opinions on pharmaceuticals are not available on the official website of health technology assessment agencies in Canada or Australia*.

### Research Methods

#### Nvivo Qualitative Analysis

This study used the Nvivo software to code the reports and systematically present trends in the focus of clinical evidence and economic models found in different country reports. NVivo is one of the computer-assisted qualitative data analysis software systems (CAQDAS) developed by QSR International (Melbourne, Australia), the world's largest qualitative research software developer. This software allows for qualitative inquiry beyond coding, sorting, and retrieval of data. It was also designed to integrate coding with qualitative linking, shaping, and modeling (29). By importing the queried assessment reports into Nvivo 12.0, the reports in the file were coded correspondingly (Appendix Table 1) after the creation of the node system (Appendix Figures 1A–C), and the criteria present in the access decisions in the four countries in terms of clinical evidence were explored through the case node classification and node matrix.

#### SPSS Consistency Analysis and Correspondence Analysis

The coded nodes were exported to an Excel spreadsheet, and the data were entered with the help of SPSS 20.0. A kappa consistency and correspondence analyses were performed for the HTA of orphan drugs in four countries. In this paper, the secondary nodes “clinical trials,” “clinical endpoint,” and “economic models” under the primary node “clinical evidence” were selected. By analyzing the correspondence between the country or recommendation situation and the assessment evidence, a scatter plot distribution between the country or recommendation situation and each node element is presented. This analysis was done to analyze the preference of countries by vector distance, specifically by connecting a line from the center (0.0) to any point and extending it in the reverse direction, and then drawing the other points to this line and its extension to make a vertical line. The closer the vertical point is to the positive vector direction, indicates the closer the relationship is, and the preference can be ranked.

## Results

### Consistency Analysis of the Four Countries' Assessments

The values in the Table 2 are Kappa scores with progressively higher concordance ranging from −1 to 1 during the consistency testing of these 15 drugs. This time, only three other national technology assessments were tested for consistency with each other, and the results are shown in Table 2. The data in the table show a poor consistency between England, Scotland, and Canada (−0.410 < kappa score < 0.192), but in comparison, the consistency between England and Scotland is stronger.

**Table 2:** Kappa consistency test scores for the four country assessments.

	**England**	**Scotland**	**Canada**	**Australia**
England	—	0.192	−0.410	*
Scotland		—	−0.320	*
Canada			—	*
Australia				—

The column “^*^” is not in line with the Kappa test conditions.

### Analysis of the Main Influencing Factors in Each Country

#### Correspondence Analysis Between Country and Clinical Evidence

**Figure 1:**
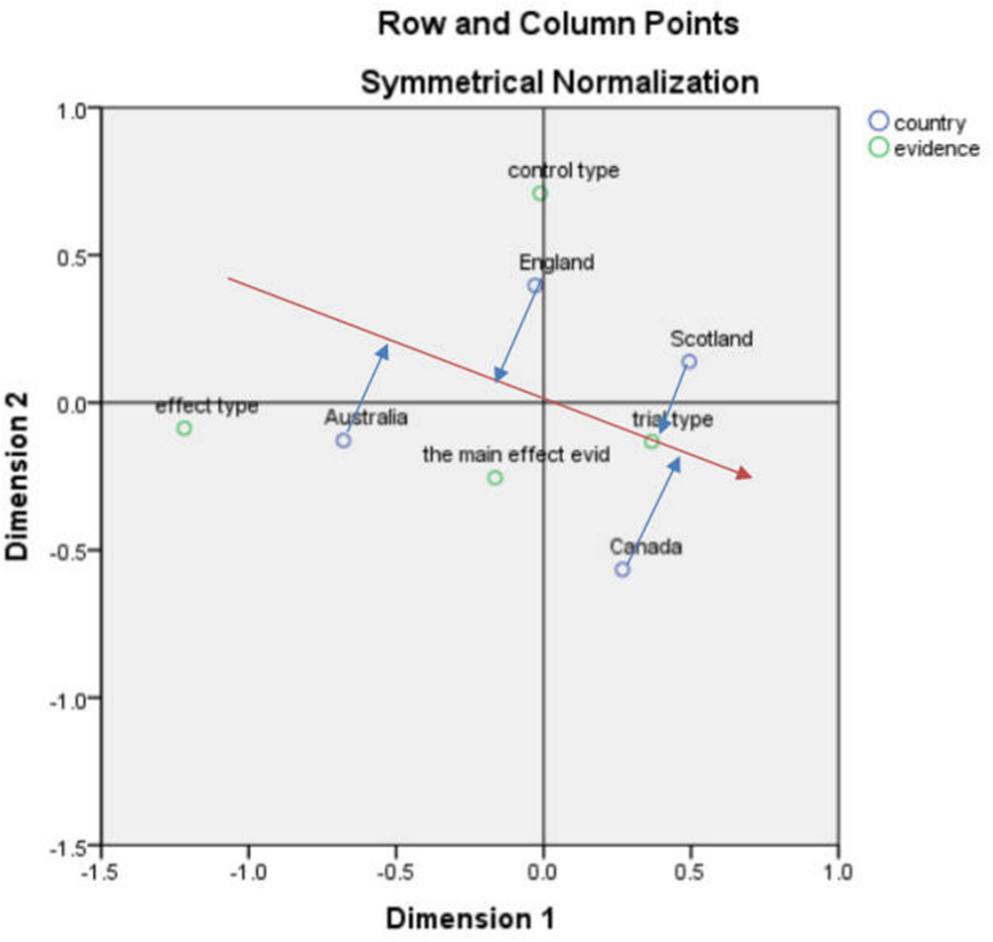
Correspondence analysis biplot (country and clinical evidence).

After performing the corresponding analysis of national and clinical evidence, the SPSS result indicated that the first and second dimensions carried 89% of the information with a chi-square value of 10.436, *p* = 0.316 > 0.05, meaning that the association was not significant (Figure 1).

According to the vector analysis method, using the “trial type” in Figure 1 as an example, a vector from the center to the “trial type” was drawn and then a vertical line (blue line) from England, Scotland, Australia, and Canada to this line and the extension line (red line) was drawn. Scotland's distance to the vertical line was closer to the positive vector direction, indicating that Scotland is more concerned about the “trial type”. The other results are analyzed in the same way to roughly derive the main concerns of each country.

Each of the four countries has its own focus on the process of health technology assessment with England being strongly associated with the control type, indicating that England focuses more on the control type. Australia focuses more on the effect type followed by the assessment of the main effect evidence. Canada focuses more on the trial type followed by Scotland. In terms of the control type, the emphasis extended from highest to lowest in England, Scotland, Australia, and Canada, while the four countries with a higher emphasis on the effect type were Australia and England.

#### Correspondence Analysis Between Country and Economic Model

**Figure 2:**
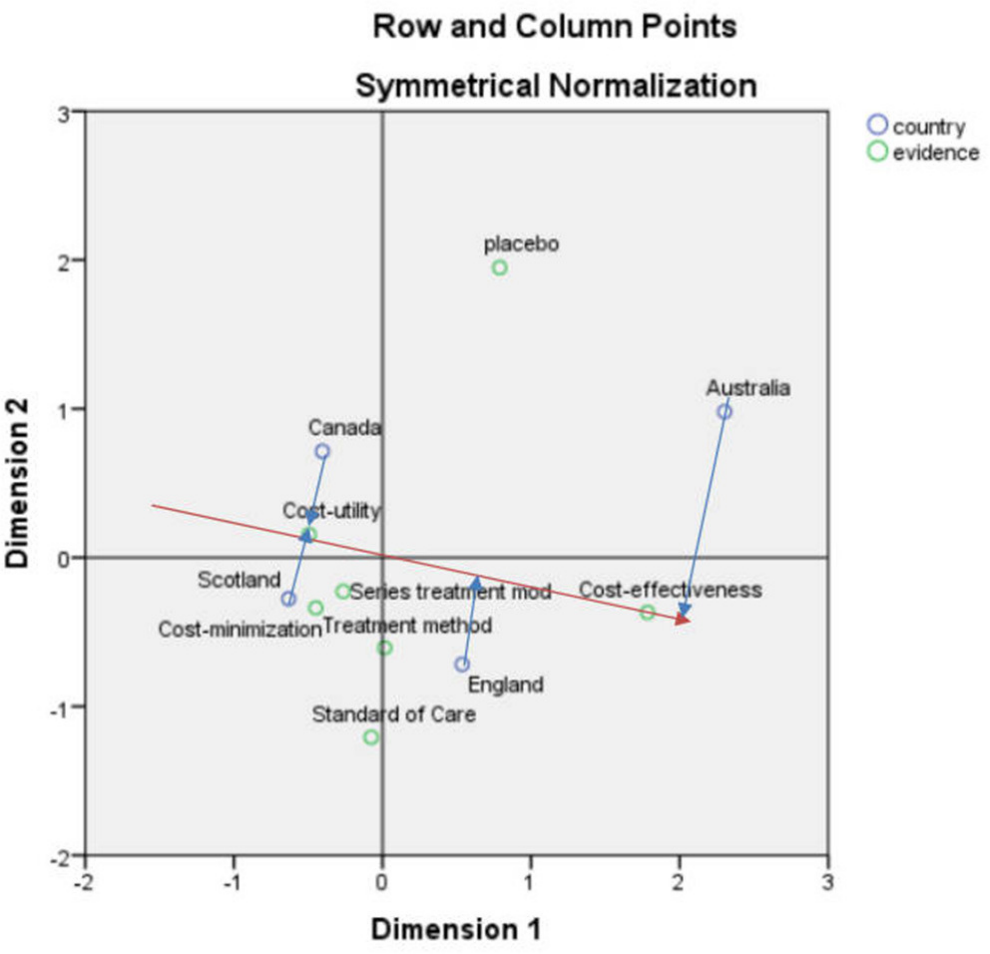
Scatterplot of country and economic model correspondence analysis.

After the corresponding analysis of the country and economic model, the SPSS result indicates that the first and second dimensions carry 97.4% of the information, and the chi-square value was 25.038, *p* = 0.124 > 0.05, showing that the association was not significant (Figure 2).

According to the vector analysis method, “cost-effectiveness analysis” in Figure 2 was used as an example, and a vector from the center to the “cost-effectiveness analysis” was constructed after which a vertical line (blue line) from England, Scotland, Australia, and Canada to this line and its extension (red line) was drawn. The vertical line corresponding to Australia was closer to the positive vector direction, indicating that Australia is more concerned with “cost-effectiveness analysis”

After performing other vector distance analyses, it can be seen that in the analysis of health technology assessment reports in the four countries, Canada focuses most on the assessment of cost-utility and cost-minimization analyses. Scotland is closely related to many elements in the assessment process, including cost-minimization analysis, series of treatment models, and cost-utility analysis. England and Australia focus on cost-effectiveness and for cost-utility analyses, and the emphasis is on the order of Canada, Scotland, England, and Australia.

#### Correspondence Analysis Between Recommendation and Clinical Evidence

**Figure 3:**
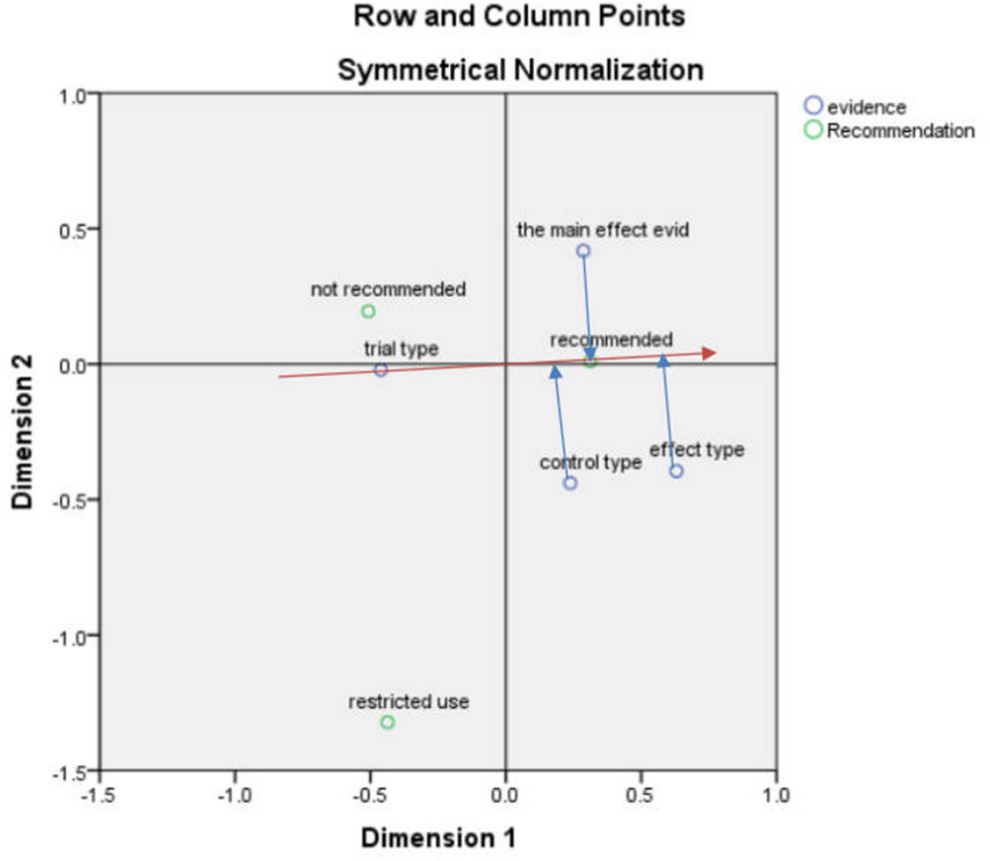
Correspondence analysis biplot (recommendation and clinical evidence).

After obtaining the correspondence analysis of the recommendation situation and clinical evidence, the SPSS result indicated that the first and second dimensions carried nearly 100% of the information, and the chi-square value was 6.621, *p* = 0.357 > 0.05, indicating that the association was not significant (Figure 3).

According to the vector analysis method, taking the “recommendation” in Figure 3 as an example, the vector was constructed from the center to the “recommendation”, and then the “effect type”, “main effect evidence” and “control type” were made to this straight line and the extension line (red line) to make a vertical line (blue line). The vertical line made by the “effect type” was closer to the vector positive direction, which indicated the “effect type” was closer to the “recommendation”.

Among the three types of recommendation, the recommendation focused on the effect type, evidence of main effects, and control type in order, which were optimized to facilitate good results in the HTA evaluation of drugs, The non-recommendation and restriction types focused more on the evaluation of the trial type, which may indicate that the trial type influences the national recommendation of a drug when it is evaluated.

#### Correspondence Analysis Between Recommendation and Economic Model

**Figure 4:**
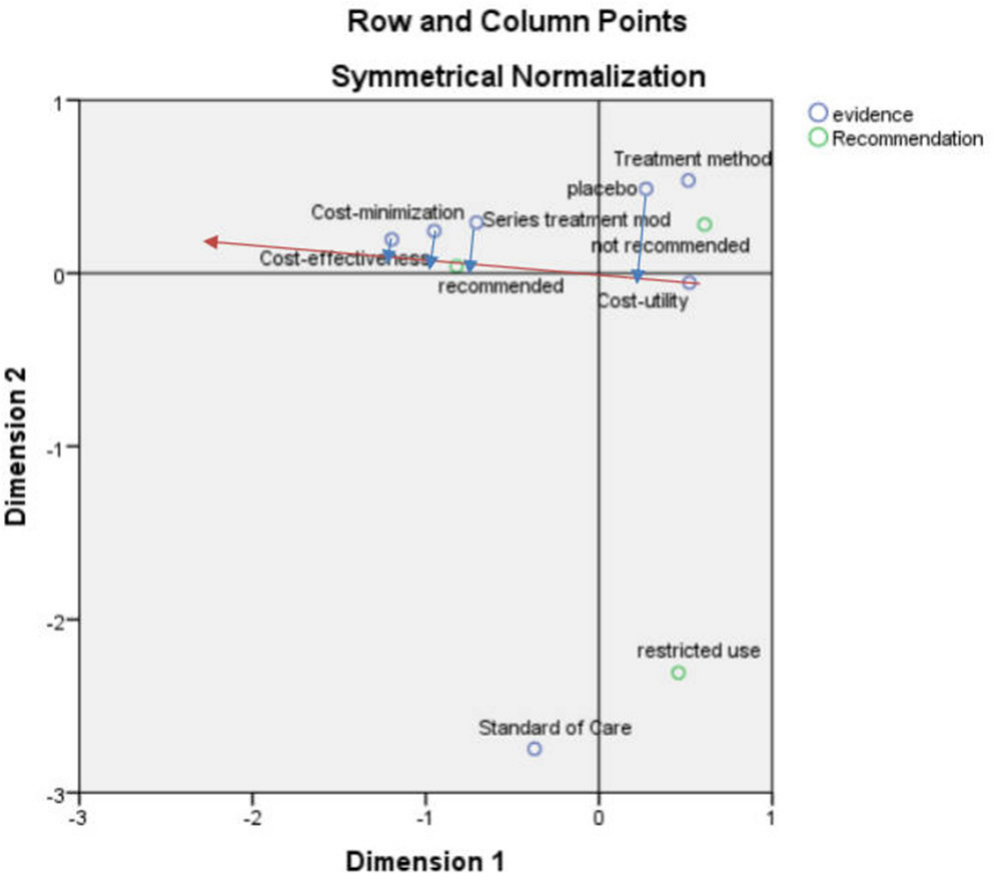
Correspondence analysis biplot (recommended scenarios and economic model).

After the correspondence analysis of the recommended and economic models, the SPSS result indicated that the first and second dimensions carried nearly 100% of the information with a chi-square value of 17.555, *p* = 0.130 > 0.05, showing that the association was not significant (Figure 4).

According to the vector analysis method, taking the “recommendation” in Figure 4 as an example, the vector was constructed from the center to the “recommendation” after which the “placebo,” “series of treatment models,” “cost-minimization analysis” and “cost-effectiveness analysis” were drawn to this straight line and the extension line (red line) to the vertical line (blue line). The vertical line formed by the “cost-effectiveness analysis” was closer to the positive vector direction, indicating that the “cost-effectiveness analysis” was closer to the “recommendation”.

According to the scatter plot (Figure 4), the recommendation focused on the elements of cost-effectiveness and cost-minimization analyses, serial of treatment models, standard of care, and placebo in that order, which can be considered to have some influence on the recommendation, but most focus was on cost-effectiveness analysis. The non-recommendation focused more on treatment method, cost-utility analysis, and placebo, and the restricted recommendation focused more on standard of care.

### Case Analysis

To present a more intuitive picture of the process of forming opinions on drug reviews by national health technology assessment bodies, the drug assessment process can be outlined by means of a case study, which can then be combined with the above conclusions for the purpose of promoting understanding or mutual argumentation. The subject of this case study was Obinutuzumab.

#### Introduction to Obinutuzumab

Rituximab was first approved by the United States Food and Drug Administration (FDA) on November 26, 1997 for the treatment of relapsed or refractory non-Hodgkin's lymphoma (NHL). As the concept of rituximab resistance emerged, in this context, scientists developed Obinutuzumab as a way to compensate for the lack of rituximab. Obinutuzumab is a novel, humanized, type II glycoengineered monoclonal antibody (mAb) directed against the CD20 antigen found on the surface of most malignant and benign B-cell-derived cells. The glycoengineering process used in the development of this agent increases its anti-lymphoma activity by enhancing binding affinity to the FcγRIII receptor on immune effector cells (30).

#### Obinutuzumab Recommendations by Four Countries

Roche Pharmaceuticals submitted applications for evaluation of Obinutuzumab to four countries in 2016–2017 and the HTAs and agencies in each of the four countries gave different evaluations. Overall, the four countries analyzed the value of the drug in terms of clinical effectiveness, safety, and cost-effectiveness to reach a conclusion on whether to recommend the drug.

Scotland explicitly does not recommend the drug, whereas Australia and Canada both recommend it. England also recommended the drug although with two conditions: (1) a Follicular Lymphoma International Prognostic Index (FLIPI) score >2 in patients with follicular lymphoma and (2) the company offered Obinutuzumab at the same discount as agreed in the patient access program.

#### Analysis of the Four-Country Assessment Process

Throughout the health assessment reports from the four countries, the first common point of assessment that was addressed was drug effectiveness. The clinical evidence from Australia, Scotland, and the UK all came from the same trial, GALLIUM, while the clinical evidence from Canada came primarily from another clinical trial, GADOLIN. The clinical evidence from the GALLIUM trial is representative of the three countries and meets the requirements for testing the clinical effectiveness of Obinutuzumab. The Australian evaluators concluded that Obinutuzumab was significantly better than rituximab in terms of progression-free survival (PFS). The Canadian evaluators based clinically meaningful improvements in PFS and overall survival (OS) while under treatment with Obinutuzumab and the net clinical benefit of the drug in combination with bendamustine. Scotland verified obinutuzumab's good clinical performance in terms of PFS while England concluded that Obinutuzumab delayed disease progression in the short-term, but its long-term impact on PFS is uncertain.

Similar to clinical outcomes were drug safety studies, which were given more attention in all four countries. The Australian evaluation found more patients with renal function-related adverse events and toxic effects (AEs) while taking Obinutuzumab. The Canadian evaluation noted a higher frequency of serious AEs in the Obinutuzumab combination group. The Scottish safety evaluation concluded that because Obinutuzumab was used concurrently with chemotherapy during the induction phase, it was difficult to fully determine the overall impact of AEs due solely to Obinutuzumab. England had similar findings to the first three with obinutuzumab-based therapy having a higher incidence of AEs when compared with rituximab-based therapy.

In addition to the aforementioned efficacy and safety, the cost-effectiveness of the drug was also highlighted in the four countries' evaluations. While Canada, Scotland, and England all reported extensively on the cost-effectiveness of Obinutuzumab, Australia did not mention it throughout the report, consistent with the findings from the corresponding analysis above. Canada did not consider the uncertainty of the incremental effect of Obinutuzumab in the economic model but rather placed great importance on the assessment model, the selection of indicators, and the reasonableness of the data in the cost-effectiveness assessment. A key factor in Scotland's refusal to recommend the drug for use in the country was the lack of stability in the cost- effectiveness analysis model submitted by the applicant company. England, while also acknowledging the immaturity of the duration of treatment effect and clinical data in the model analysis, also felt that significant uncertainty in the evidence base and incremental cost effectiveness ratio existed. However, the Committee concluded that Obinutuzumab was a cost-effective approach to the use of NHS resources in England for patients with untreated follicular lymphoma (FLIPI score >2).

The four countries possess some homogeneity among their assessments, but some particularities in certain countries could be noted. The most notable feature of the Australian assessment body is the two rounds of risk-benefit assessment that it conducts, which aims to achieve optimal treatment outcomes while controlling treatment risks. In addition, Canada has a pharmacovigilance in addition to a risk management program designed to identify identifiable and potential risks and missing information to ensure safe use of medications. One element of the Scottish assessment process is novel in that it sought the views of physicians and patients who use the drug in order to consider the added value of Obinutuzumab as an orphan drug in the context of existing treatment in the NHS in Scotland. England's assessment also considered the innovative nature of the drug with the committee hearing from clinical experts that Obinutuzumab has a similar mechanism to rituximab and that Obinutuzumab is not an innovative drug.

## Discussion

Of the 15 drugs studied above, Australia was found to have a recommended status for the nine drugs for which assessments were available with a single assessment that did not qualify for Kappa analysis and therefore could not be tested for consistency between Australia and the other countries. Canada and England had only recommendation and non-recommendation statuses, while Scotland also had a restricted status, which allowed for better application of drugs to achieve better symptomatic use and reduce adverse effects of drugs. The four countries differed in their preferences for assessing clinical evidence and the economic model for orphan drugs. England placed more emphasis on treatments in terms of control type than did the other countries, Canada had a greater proportion of Phase III and open-label trial types and focused more on cost-utility analysis, while Australia was less studied in terms of economic model, probably because of the different review processes in these four countries. In Australia, the PBAC's primary role is to recommend which medicines should be subsidized, and the Australian government determines the final listing decision (31). The NHS in England must comply with positive recommendations by NICE, a process that resembles the Scottish NHS boards. These differences may explain why less attention has been paid to the economic modeling aspect in Australia.

For national clinical trials, England and Scotland focus more on the assessment of clinical trial directions, while Canada and Australia focus on the assessment of clinical endpoints. PBAC has a clear preference for head-to-head RCT (if available) and indirect comparisons (32), while placebo comparisons are listed as acceptable evidence for NICE and SMC (5), the strength of evidence required for clinical trials vary to different countries. In the analysis of clinical uncertainty, it can also be seen that uncertainty in evidence and study design occupies most of the English and Scottish evaluation reports followed by clinical effectiveness, indicating that these two countries are likely to focus on these two aspects of uncertainty when conducting their evaluations. Their processes precisely indicate the focus and difficulty of addressing orphan drugs in health evaluation and needs more attention.

The case study demonstrates that although Scotland affirmed the good clinical performance of Obinutuzumab in PFS, it still refused to recommend the drug for reimbursement in this country due to the lack of stability of the submitted cost-effectiveness analysis model. In contrast, England recommended the drug for reimbursement by limiting its indications and taking advantage of discounts agreed upon the patient access scheme. The decision making in Canada and Australia was focused on the clinical benefit of the drug and neglected to assess the uncertainty of the economic benefit. This finding also matched the results of the analysis in the article by Nicod (5) in which the SMC seems less likely to approve drugs with high and uncertain clinical cost-effectiveness and the CDR seems to place more emphasis on the relative effectiveness of new treatments among others.

The poor consistency of the four national assessments may be due to the fact that the focus of the assessment of orphan drugs in different countries is based on the prevalence of rare diseases in their countries, and that the efficacy requirements for orphan drugs may differ from country to country, with each country focusing on the criteria of the actual development in their country when conducting the assessment. Selection of study subjects is a component of health technology assessment report with variability in the review opinions of the four countries. The differences may also be due to the existence of manual coding errors and the limitations of the information obtained from the 15 orphan drug reports that were selected. In future studies, the sample size should be enlarged, and more representative countries should be selected to extrapolate the findings.

## Actionable Recommendations

Due to the specificity of rare diseases, clinical trials have a small subject population, and the efficacy is often uncertain while the effectiveness and efficacy of drugs are factors of great concern to countries with respect to the HTAs of orphan drugs. The uncertainty of efficacy will lead to an increase in the instability of pharmacoeconomic models, thus making the results of cost effectiveness of drugs unstable and often not cost effective due to the high price of orphan drugs, which eventually leads to orphan drugs not being recommended to the health insurance list of each country or strictly limited for certain indications.

Countries have different levels of economic development and social conditions, such as population, and large uncertainties in their clinical evidence and economic evidence exists, which brings challenges to the HTA review process in each country. Currently, no uniform standards in the review of orphan drugs exist, so countries show differences in their attention to different types of evidence when making decisions. In the process of HTA of orphan drugs, the analysis should not be limited to cost effectiveness but also evaluate various factors, such as the innovativeness of orphan drugs, the increase in the utility of family members, and the improvement of life satisfaction when treating them on the basis of the sustainability of health insurance funds and through the collection of real-world data, in order to enhance the accessibility of drugs for patients. In addition, the uncertainty of the clinical use of orphan drugs through real-world data collection can be reduced and the economics of orphan drugs not only through NHS reimbursement but also through multi-channel economic sharing mechanisms could be improved, so that more patients with rare diseases can be treated and their economic burden can be effectively alleviated through price agreements or joint multi-level protection.

For example, in England, ICERs for common drugs must meet the threshold of £20,000–30,000 per QALY to enter the NHS system, but the majority of ICERs for orphan drugs are higher than the upper threshold of willingness to pay set by England, and NICE has introduced social value judgments to evaluate orphan drugs, namely combining the social benefits of orphan drugs, innovation, the number of people with the rare disease, the specificity of the disease condition, and others to decide whether to include them in the NHS system (33). In addition, NICE conducted a highly specialized technology evaluation (HSTE) for high-value drugs (ultra-rare drugs) with a willingness-to-pay threshold adjusted to cost £100,000 per QALY obtained. It is also clear from the case studies that in response to the uncertainty of the long-term effects of orphan drug efficacy and the instability of economic models, England has saved some patients first by entering into price agreements and then collecting evidence and revising the results in subsequent evaluations.

Currently, the focus of HTA development is very different among different countries, and different countries have different degrees of orphan drug coverage and different goals when making HTA decisions for orphan drugs. A need to fix the HTA shortcomings, build a more mature assessment mechanism, and explore a more complete and appropriate reimbursement pathway in the field of rare diseases exists with a view to providing a basis for health decision-making. Concurrently, exploring the use of real-world data for orphan drug access and security and making good use of HTA to promote more marketing and inclusion of orphan drugs in health insurance combined with the country's own development, can guarantee the safety and accessibility of orphan drugs, and actively safeguard the rights of patients with rare diseases.

## Author Contributions

NZ carried out the search and selection of the articles and drafted the manuscript. HJ was involved in the design of the study and critically reviewed the manuscript. ZL participated in data analysis and literature review. JH gave guidance on the logic and key problem of the article. J-HX participated in data analysis. Y-HF performed the literature searches. NY was involved in the design of the study, conceived of the study, and critically reviewed the manuscript. All authors contributed to the article and approved the submitted version.

## Conflict of Interest

The authors declare that the research was conducted in the absence of any commercial or financial relationships that could be construed as a potential conflict of interest.

## Publisher's Note

All claims expressed in this article are solely those of the authors and do not necessarily represent those of their affiliated organizations, or those of the publisher, the editors and the reviewers. Any product that may be evaluated in this article, or claim that may be made by its manufacturer, is not guaranteed or endorsed by the publisher.
