# Supplementary material for: Influencing Factors of Health Technology Assessment to Orphan Drugs: Empirical Evidence in England, Scotland, Canada, and Australia
Source: Front Public Health. 2022 Jun 17;10:861067. doi: 10.3389/fpubh.2022.861067 (PMC9247336; doi:10.3389/fpubh.2022.861067)
Supplement: Supplementary file 1 [file Table_1.DOCX]

Appendix Table 1: HTA assessment report node system(partial)

| Name | Files | References |
| --- | --- | --- |
| 1 Clinical trials | - | - |
| 1.1Trial Type | - | - |
| 1.1.1 Phase III | 19 | 21 |
| 1.1.2 Phase II | 11 | 15 |
| 1.1.3 Extended trial | 0 | 0 |
| 1.1.4 Open label trial | 22 | 26 |
| 1.1.5 Indirect comparison | 16 | 17 |
| 1.1.6 Subgroup analysis trials | 3 | 3 |
| 1.2 Main trial evidence | - | - |
| 1.2.1 Main trial | 1 | 1 |
| 1.3 Control Type | - | - |
| 1.3.1 Placebo | 8 | 8 |
| 1.3.2 Treatment method | 21 | 24 |
| 1.3.3 Standard of care | 2 | 3 |
| 1.3.4 Standard of care-placebo | 2 | 2 |
| 1.3.5 None | 1 | 1 |
| 2 Clinical endpoint | - | - |
| 2.1 Effect type | - | - |
| 2.1.1 Clinical endpoints | 12 | 13 |
| 2.1.2 Proxy endpoints | 0 | 0 |
| 2.1.3 Health-related quality of life | 1 | 1 |
| 2.1.4 Not specified endpoint | 2 | 2 |
| 2.2 Evidence of main effects | - | - |
| 2.2.1 Main endpoint | 28 | 35 |
| 2.2.2 Secondary endpoint | 21 | 27 |
| 3 Economic model | - | - |
| 3.1 Types of economic models | - | - |
| 3.1.1 Cost-utility analysis | 20 | 25 |
| 3.1.2 Cost-effectiveness analysis | 6 | 6 |
| 3.1.3 Cost minimization Analysis | 4 | 4 |
| 3.2 Economic model control | - | - |
| 3.2.1 Placebo | 2 | 3 |
| 3.2.2 Treatment method | 4 | 4 |
| 3.2.3 Standard of Care | 2 | 3 |
| 3.2.4 Series of treatment models | 3 | 3 |
