# Supplementary figures and images for: Influencing Factors of Health Technology Assessment to Orphan Drugs: Empirical Evidence in England, Scotland, Canada, and Australia
Source: Front Public Health. 2022 Jun 17;10:861067. doi: 10.3389/fpubh.2022.861067 (PMC9247336; doi:10.3389/fpubh.2022.861067)

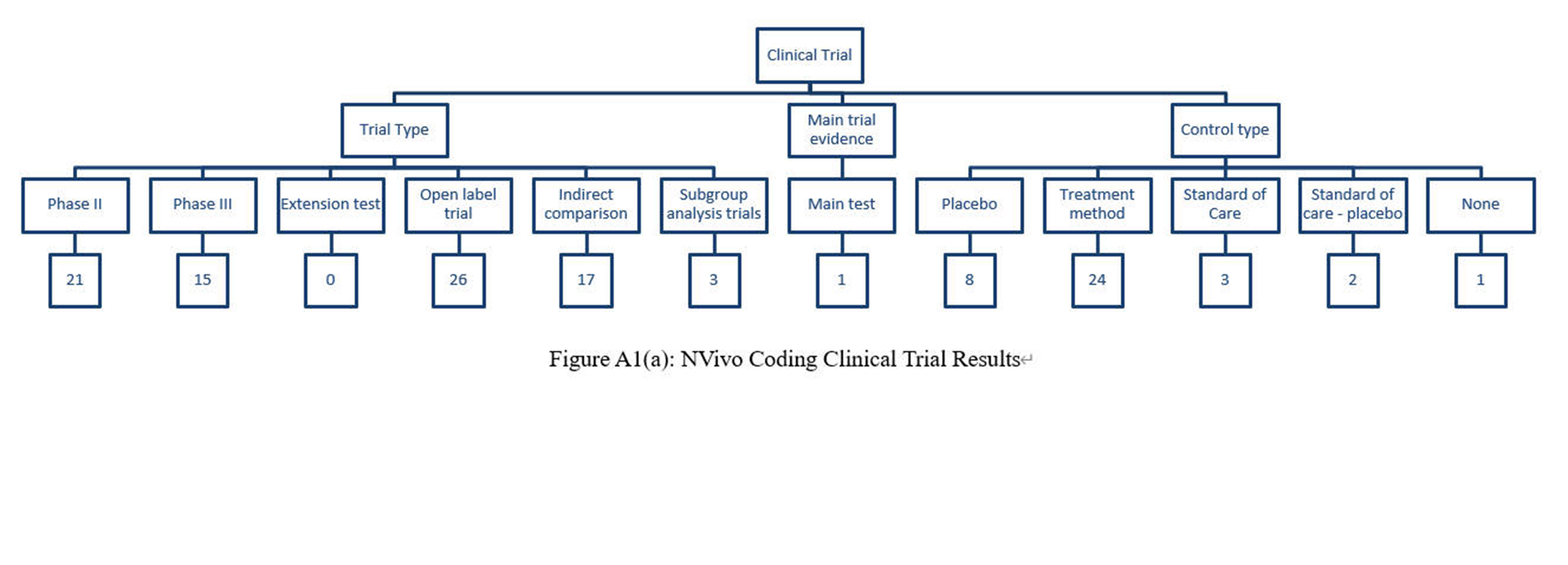

Supplement: Supplementary file 2 [file Image_1.JPG]

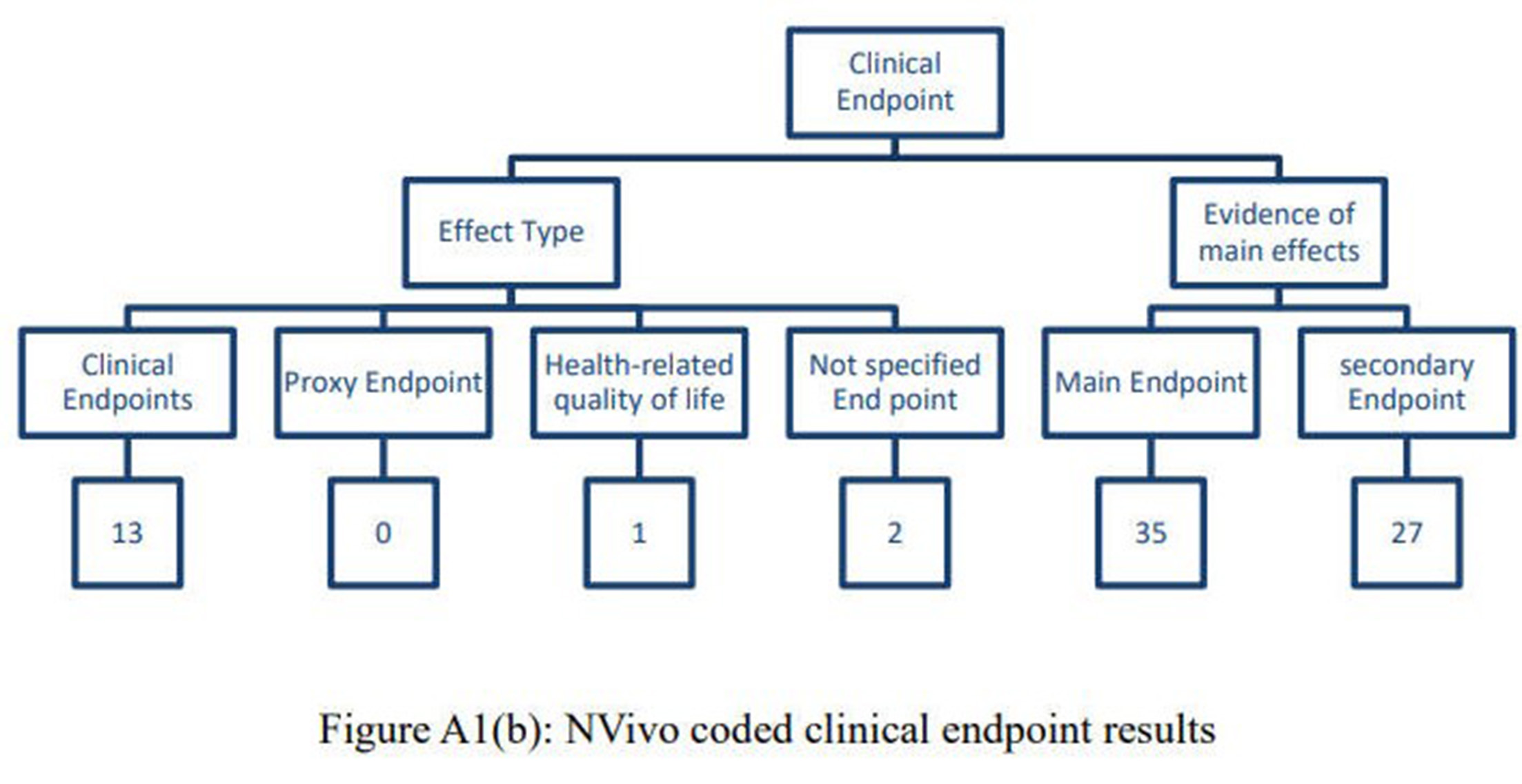

Supplement: Supplementary file 3 [file Image_2.JPG]

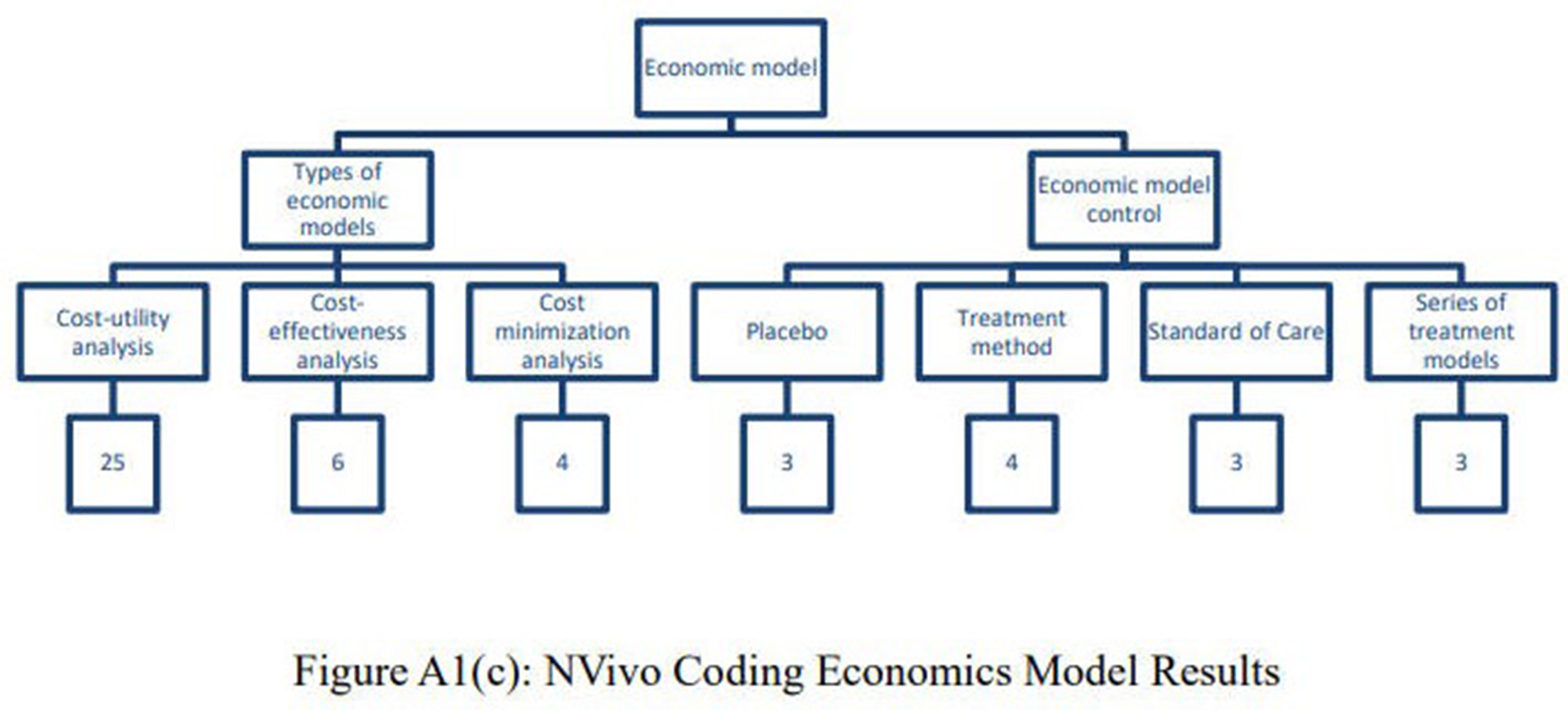

Supplement: Supplementary file 4 [file Image_3.JPG]
